# Avian Flu: Examining Role of Ducks in Indian Context

**DOI:** 10.4103/0970-0218.66854

**Published:** 2010-04

**Authors:** Rajan R Patil

**Affiliations:** Division of Epidemiology, School of Public Health, SRM University, Chennai, India

The research work carried out in Vietnam and Thailand has the potential to completely change our approach to the bird flu control. There is an increased understanding of avian flu transmission and its control nowadays. The GIS-Epidemiological work done in South East Asian countries implicates ducks as the main source of bird flu outbreaks.(1) Ducks-rice fields-human interaction is now recognized as responsible for the bird flu flare ups in Asian countries. The present study takes into consideration this interaction as well as a set of five key environmental variables comprising elevation, human population, chicken numbers, duck numbers, and rice cropping intensity for three synchronous epidemic waves in Thailand and Vietnam. A consistent pattern emerges, suggesting that risk is significantly associated with duck abundance, human population, and rice cropping intensity in contrast to a relatively low association with chicken numbers. It is interesting to note that the study downplays chicken as the main source of bird flu. It unequivocally says that mass vaccination of chicken is ineffective in controlling bird flu in countries like Vietnam.

This study is an attempt to discuss the above findings in the Indian context. First, we need to take these findings cautiously as these studies are performed in the focal geo-ecological setting of Vietnam, and therefore the findings may vary in Indian context. If we attempt a cursory extrapolation of these findings to Indian avian flu outbreaks, ecologically at least, we could relate the West Bengal outbreaks to the duck-rice field-population theory. West Bengal is one of the top rice growing states in India and culturally most of the rural. WB rural households do have small ponds and rear ducks in their backyard. In the case of Maharashtra bird flu outbreak, the duck-rice field theory does not hold good, as the pieces of puzzle do not fit in the case of Nandurbar and Jalgaon districts in Maharashtra where the first and subsequent outbreak of bird flu were reported from India. These regions are dry and obviously rice is not the main crop here. Moreover, these regions are famous for banana plantations (Unlike West Bengal which leads in rice production, Maharastra is known for its horticulture prowess-mainly the production of mangoes, oranges, grapes, etc). Other major crops are cotton and sugarcane. Duck rearing is not prevalent in Maharashtra, as they could only be reared in watery and marshy regions.

In the poultry sector, ducks form only about 10% of the total poultry population in India and contribute about 6-7% of the total eggs produced in the country. Ducks are mostly concentrated in the Eastern and Southern States along coastal regions of the country.(2) As having a coastal line, West Bengal (an Eastern state) is ideal for duck rearing. In contrast, Maharashtra (a Western state) is not known for duck rearing, as they could only be reared in watery and marshy regions. The subsequent paragraphs deal with the distribution of chickens across India and occurrence of the bird flu outbreak and investigate any possible pattern if any. The distribution of poultry population suggests that 42% of the total population of poultry birds is confined to Southern region, 22% in the Eastern region, and 20% in Western region and the remaining 16% in the Northern region.(3)

Interestingly, the pattern shows negative correlation, that is, no bird flu is reported from areas having highest poultry concentration, whereas bird flu is reported from areas having least poultry concentration. Nearly half of the poultry come from Southern India (Andhra Pradesh is the No 1 in poultry rearing in India), yet entire region remained free from bird flu outbreak. Maharashtra state which reported first outbreak of avian flu is in the Western region which has the least concentration of poultry in India.

The rice-ducks-human interaction offers explanation for West Bengal’s vulnerability to bird flu *visa vie* as a top rice producing and duck rearing state but fails to account for Maharashtra outbreaks. Although rice-duck-human theory partially correlates with Indian bird flu scenario, it raises many new questions, for example, if rice ducks are pre-requisite, then the Southern states of Andhra Pradesh, Tamil Nadu should have been more vulnerable for their rice and duck production status along Southern Coast.

In the global scenario as well, the rice-duck-human theory appears to correlate well with South East Asian countries. These countries are the main rice producing nations in the world and incidentally avian flu originated in this region and continues to be the epicenter for avian flu. The top ten rice producing nations in the world are China, India, Indonesia, Bangladesh, Vietnam, Thailand, Myanmar, Philippines, Japan, Brazil and 90% of the world’s 1044 billion domestic ducks are in Asia, with 775 million of them (75%) are in China and Vietnam.

But how do we account for bird flu outbreaks in the European countries and other continents where neither rice nor ducks are the mainstay production? The strength of this theory is its validity and appears to have good correlation with spatial and temporal evolution of avian flu *visa vie* rice fields and duck rearing. At the same time, we should be awakened to the fact that these types of studies come under the category of ecological studies and are prone of ecological fallacy.
